# Primary health care experiences of Indian immigrants living with chronic illnesses in Australia: a qualitative study

**DOI:** 10.1017/S1463423626101339

**Published:** 2026-06-15

**Authors:** Robina Singla, Sheeja Perumbil Pathrose, Aileen Pamonag Lane, Olayide Ogunsiji

**Affiliations:** School of Nursing and Midwifery, Western Sydney University, Australia

**Keywords:** emigrants and immigrants, experiences, general practitioners, Indian, primary health care

## Abstract

**Aim:**

To explore the primary health care experiences of Indian immigrants living with chronic illnesses in Australia.

**Background:**

Primary health care is integral for managing health issues for people living with chronic illnesses. Despite Indian migrants being the largest overseas-born population in Australia to use primary health care services for their chronic illnesses, at this time, limited literature reports on their experiences in Australia.

**Methods:**

A qualitative exploratory approach was adopted for this study. Through purposive sampling, 11 participants were recruited, and data was collected using semi-structured interviews. The interviews were transcribed verbatim and thematically analysed.

**Findings:**

Three themes and six sub-themes emerged from the thematic analysis. The first theme ‘being a mix of experiences’ presented participants’ positive and negative experiences related to communication, compassion, quality of care and length of consultations. The second theme ‘facilitators to accessing primary health care services in Australia’ described the benefits of telehealth and having a common cultural background as that of the general practitioner. Meanwhile, the third theme ‘barriers to accessing primary health care services in Australia’ identified unavailability of preferred GPs and waiting times and delays for in-person appointments as barriers that hindered access to PHC services in Australia.

**Conclusion:**

Primary health care experiences of Indian immigrants with chronic illnesses in Australia were a mix of positive and negative experiences, similar to that of other immigrants. Faster accessibility and compassionate services among migrants were highlighted to be needing extra attention by way of further research and acknowledging the unique diversity of individuals.

## Introduction

Chronic illnesses are prolonged conditions lasting for at least six months, which have persistent effects on quality of life and require ongoing care and management from a multidisciplinary health care team (Australian Health Minister’s Advisory Council, 2017; Gordon *et al*., 2018). Around 41 million people die each year as a result of chronic illnesses, equating to 74% of deaths globally (World Health Organization, 2023). In 2022, 171,500 deaths (90% of all deaths) in Australia were due to chronic illnesses and around 12.6 million people (49.9%) residing in Australia for at least 12 months were living with at least one chronic condition (Australian Bureau of Statistics [ABS], 2023). Primary health care (PHC) delivers essential early interventions that facilitate prevention, early detection, and treatment by a multidisciplinary team of health care professionals for people living with chronic illnesses in the community (Bierman *et al*., 2021; World Health Organization, 2023).

The Australian health care system comprises of a public and private sector (Australian Institute of Health and Welfare [AIHW], 2025). Medicare, Australia’s universal health insurance scheme is a federal funded programme. It provides free or subsidized treatment for Australian citizens, permanent residents, and visitors from countries with reciprocal health agreements who use public hospitals, community services, and general practitioners (GPs) (AIHW, 2022). Private health insurance provides Australian citizens with the option to access private hospitals, specialists, dental care, and allied health services that are not fully covered by Medicare (AIHW, 2025). Nursing services are provided in both public and private sectors and, therefore, are funded by either Medicare or private health insurances (Department of Health, Disability and Ageing, 2025). Over the years, PHC in Australia has evolved to adapt to the changing health care needs of the diverse population, of which, over 30% was born overseas (ABS, Australian Bureau of Statistics, 2026). However, culturally and linguistically diverse immigrants with complex health care needs face challenges of accessing suitable health care and navigating Australia’s complex health system (Department of Health, 2022; Mengistu *et al*., 2023). These include language barriers (Kay *et al*., 2016), lack of recognition of cultural diversity, and limited levels of cultural competence (Harrison *et al*., 2019). Chinese immigrants, particularly the elderly and those with poor English proficiency, faced language and communication problems and had difficulties navigating the health care system and resources (Jin *et al*., 2020; Zhang *et al*., 2020). Vietnamese immigrants reported to have similar access barriers by way of language difficulties and a lack of health information available in their language (Jin *et al*., 2020). Furthermore, travel time, cultural and religious differences and affordability affected accessibility among African immigrants (Anaman-Torgbor *et al*., 2017). These issues influence poor heath service utilization and, consequently, increase the risks of unmanaged chronic health conditions.

Indian immigrants in Australia are the largest group born overseas, with a population of 916,000, and thus, the largest group to avail Australia’s PHC services (ABS, Australian Bureau of Statistics, 2026). One in three Indian immigrants in Australia has a chronic condition which is managed and treated through PHC (AIHW, 2022; Department of Foreign Affairs and Trade, 2022). People of Indian cultural background in Australia are at a higher risk of insulin resistance and have the third-highest prevalence of type 2 diabetes mellitus (Department of Foreign Affairs and Trade, 2022). Back pain, depression, hypertension and heart disease were the most reported diseases in an Australian study (Nisar *et al*., 2025). South Asian immigrants in the UK, US, Norway and Canada are at a higher risk of abdominal obesity, hypertension, dyslipidemia and impaired glucose intolerance, which directly contribute to cardiovascular disease and type 2 diabetes mellitus (Mahadevan *et al*., 2023). In America, one in four deaths among Indian immigrants is caused by cardiovascular disease (Gidwani *et al*., 2021). Deficiency in Vitamin D is also common among Indian immigrants living in countries such as the UK, US, Canada, South Africa, and Australasia (Darling, 2020; Partha, 2024). Apart from Indian immigrants being the largest immigrant group in Australia, and the largest to use PHC services to manage their chronic health conditions, they consider medical expertise, responsiveness, and inclusion in decision making processes when assessing the quality of the health care they receive (Chatterjee and Srinivasan, 2013). Communication due to socio cultural differences is reported to be a barrier in accessing health care among south Asian migrants (Adhikari *et al*., 2021; Nisar *et al*., 2022). However, the specifics for Indian immigrants in PHC is lacking in empirical research. While the above evidence explores the access and decision-making issues experienced by migrants, the current study focuses solely on the experiences of Indian immigrants with PHC services in Australia. Considering the large proportion of Indian immigrants with chronic illnesses that are managed through PHC, their cultural variations and ethno-specific healthcare needs warrant concerted information on their experiences with PHC services.

## Aim

To explore the PHC experiences of Indian immigrants living with chronic illnesses in Australia.

## Methods

### Study design and setting

A qualitative exploratory approach was adopted using virtual semi-structured interviews that facilitated in-depth exploration of personal experiences of Indian immigrants regarding PHC services across Australia, a topic that remains under-explored (Denzin and Lincoln, 2018). This approach facilitated deeper insights that form the foundation of more specific research in future. Rigour was maintained throughout the study by meeting four criteria as recommended by Hammarberg *et al*., (2016). These criteria were trustworthiness, credibility, applicability, and consistency. Trustworthiness was achieved by presenting a detailed description of the study purpose and process, including how decisions were made and how data was generated. Credibility was achieved by using robust quotes and contextual details to support the findings, along with details on reflexivity. Detailed descriptions of the sampling, recruitment, data collection and data analysis methods, and participant information are presented to achieve applicability. Additionally, presenting detailed findings that were verified by all members of the research team helped achieve consistency. The guidelines for Consolidated Criteria for Reporting Qualitative Studies (Tong *et al*., 2007) and the Consensus Reporting Items for Studies in Primary Care (CRISP) checklist (Phillips *et al*., 2023) were followed.

A social constructionism perspective guided this study. This perspective supports the notion that there are multiple realities and that one’s understanding of the world and, therefore, their perception of reality depends upon dynamic social and cultural contexts of the time (Burr and Dick, 2017; Doyle *et al*., 2019). Social constructionism has a number of assumptions including social reality being socially constructed, and reality not being an objective truth. Guided by these assumptions, we utilized semi-structured interviews that enabled participants to provide their subjective experiences of accessing PHC for the management of their chronic illnesses. Social constructionism assumes that multiple realities are possible. This informed the adequate attention that we paid to each of the participants’ interview transcripts in the course of data analysis. Even when participants provided competing and contrasting responses to the interview questions, our understanding of the social constructionism assumption that there is no single correct and factual reality guided the wide presentation of participants’ subjective quotes.

### Study participants, sampling, and recruitment

Participants were eligible for inclusion in this study if they (1) identified as Indian immigrants in Australia, (2) were aged 18 years and over, (3) had an existing chronic condition that had been diagnosed for at least 6 months, for which they were utilizing PHC services, and (4) were willing to participate. The exclusion criteria for this study were people who could not converse in the English language and people who self-reported with any cognitive impairment that would prevent them from providing informed consent and, thus, participating in the interview.

Purposive sampling was used to intentionally recruit participants who met the inclusion criteria. Self-identified adult Indian immigrants with chronic illnesses were considered central to obtaining an in-depth understanding of their lived experiences.

Participants were informed of the study using a flyer that was posted on unpaid social media platforms, including personal Facebook and WhatsApp community groups between April and May 2024. These platforms have been successfully used in previous studies to recruit research participants from diverse communities and geographical areas efficiently (Darko *et al*., 2022; Pathak *et al*., 2025). Examples of Facebook groups used in this study included ‘Indian Mums of Australia’, ‘Indian Families Connect (Sydney)’, ‘Blacktown Indian Community – Sydney’, and ‘Penrith LGA Indian Community Group’. These groups provide members with opportunities to reach out for help, support, and advice, and allow for social and community connections and networking. The first author (RS) is an Indian migrant in Australia and a member of these groups (not an administrator). There are a few similar WhatsApp groups of which the researcher is a part. These provide more localized connections and ease of access to a diverse range of potential participants. Such groups include ‘Glenmore Park Mums’ Group’ and ‘Penrith LGA Unity Group’. However, despite using social media recruitment, this did not result in a favourable outcome, perhaps due to the sensitive nature of the topic.

It was anticipated that interviewing 10 to 15 participants would provide adequate data to identify common patterns and recurring themes to gain deeper insights into Indian immigrants’ experiences with PHC services. However, data collection was stopped after engaging 11 participants, as no new concepts were identified for this exploratory study (Vasileiou *et al*., 2018; Braun and Clarke, 2022;). A participant information sheet detailing the aims of the study, data collection method, confidentiality, and voluntary participation and withdrawal was provided to interested participants. They were also provided with an informed consent form which they were asked to read, understand, and sign prior to participation. They were able to seek any clarification and ask further questions before providing their written consent and taking part in an individual interview. Before the commencement of the interview, another opportunity was provided to participants for any questions or clarifications. Their consent and willingness to participate and have their unidentifiable data used in this and further related research and publication was confirmed. They were reinformed that they could withdraw at any stage and/or refuse to answer any questions, without repercussion. No incentives were provided to participants.

### Data collection

Data for this study was collected through semi-structured interviews conducted and recorded virtually via Zoom. This was due to the convenience that remote interviews provided to participants and to ensure the comfort of a familiar environment, potentially allowing them to express their thoughts more easily (Pocock *et al*., 2021; Taherdoost, 2023). Participants were given the option to turn their video off prior to the interview and all the interviews were audio recorded. These interviews were conducted by the first author (RS), a registered nurse and researcher working in PHC. The researcher received training in qualitative interviewing techniques. To ensure methodological rigour and interviewer confidence, the first interview was conducted jointly with an experienced qualitative researcher from the team. Participants were required to provide pseudonyms to which they were referred to in this study. An interview guide with open-ended questions was utilized (Appendix A). This guide was informed by the expertise and experience of the research team and after examining existing literature while ensuring that the questions addressed the research topic. The semi-structured interviews ranged from 30 minutes to one hour, allowing for a relatively set agenda and predetermined questions while permitting probing and clarification and hence, facilitating the collection of rich data (Taherdoost, 2023). Participants were asked to share their experiences regarding the PHC services they used for the management of their illnesses. The interviewer took brief notes during the interviews, followed by detailed contextual descriptions and reflections from the interviews. The recorded interviews were transcribed verbatim, and data was stored securely in password-protected documents and folders on a secure file-sharing platform on the university’s network (*Privacy and Personal Information Protection Act 1998 (NSW), pt 2, div 1*; Western Sydney University, 2022; National Health and Medical Research Council, 2023).

### Data analysis

Data analysis was commenced simultaneously with data collection to ensure adequacy of data until no new concepts were introduced (Braun and Clarke, 2022; Klem *et al*., 2022a). Analysis of the data from the transcribed interviews was guided by Braun and Clarke’s (2023) six phases of thematic analysis of qualitative data, namely, phase one: familiarization with the data; phase two: generating initial codes; phase three: generating themes; phase four: reviewing potential themes; phase five: defining and naming themes; and phase six: producing the report. The first phase involved the first author (RS) becoming familiar with the data to identify similarities and differences across the dataset. Then, initial codes were generated before descriptive themes and subthemes were identified, reviewed, defined, and named by the research team (RS, OO, SP, AL). The themes and subthemes reflected the participants’ experiences as understood and collectively interpreted by the research team. This non-linear and iterative process was documented in NVivo 14 (Lumivero, 2023) to track the evolution of the codes and their prospective themes by the research team, thereby facilitating a transparent process of data analysis (Byrne, 2022; Braun and Clarke, 2023).

Additionally, debriefing during weekly meetings, and maintaining an audit trail with a detailed log of the research processes and decisions was maintained (Cope, 2014; Klem *et al*., 2022b). This included records of the research process such as interview transcripts, stages of data analysis, and draft reports. Furthermore, rich, vivid, and verbatim participant quotations were used to confirm that the analysis presented was collated directly from the collected data. Member checking was not used in this study as the research team felt that revisiting the sensitive areas of chronic illnesses and PHC experiences and the potential reporting of negative experiences may be triggering.

### Researcher reflexivity

It is important to acknowledge that the first author (RS) who conducted interviews of participants, is an Indian immigrant and a registered nurse working in PHC, positioning her as an insider with shared experiences to those of the participants. Of the other researchers, SP is also an Indian immigrant, and all (SP, OO, and AL) are registered nurses who have had experiences caring for Indian immigrants. As such, it was imperative to address the role, biases, assumptions, prejudices, beliefs, attitudes, and learning of the research topic to eliminate potential bias in the design of the study, recruitment of participants, collection and interpretation of data, and writing of the findings (Berger, 2015; Teh and Lek, 2018). This was achieved via a reflective journal and weekly debriefing meetings held by the research team where perspectives, interpretations and assumptions from the interviews were critically reflected upon to ensure credibility throughout the duration of this study.

### Ethical considerations

This study was approved by the institutional Human Research Ethics Committee (Approval Number: H15813).

## Findings

Eleven participants took part in this study. Their ages ranged from 24 to 51 years and had lived in New South Wales, Australia from seven to 32 years. Details of the characteristics of participants are outlined in Table 1.

**Table 1.** Characteristics of Participants (*n* =11) Table 1 long description.

Participant pseudonym	Age (years)	Gender	Years in Australia	Education background	Employment status	Chronic illness(es) & (time since diagnosis (years))	Primary health care service
Bindu	39	Female	17	Bachelors	Full-time	Endometriosis (1.5), chronic back pain (3–4), thyroid issues (7)	GP
Bree	51	Female	10	Masters, PhD candidate	Full-time	High blood pressure (5)	GP
Dal	45	Female	10	Bachelors	Part-time	Type 2 diabetes mellitus (T2DM) (4)	GP, dietitian
Nee	38	Female	10	Masters	Full-time	T2DM (5), high blood pressure (5), Polycystic ovarian syndrome (PCOS) (5)	GP, dietitian
Pur	24	Male	20	Bachelors	Full-time	Asthma (22), mental health (OCD, depression, anxiety) (5)	GP
Raul	40	Male	7	Bachelors	Full-time	Proteinuria (10–12)	GP, dietitian
Roi	32	Female	30	Bachelors	Full-time	Chronic pain (2)	GP
Sandy	39	Male	10	Bachelors	Full-time	Migraine (5)	GP
Shaf	35	Female	7	Masters	Full-time	PCOS (8–9)	GP
Victor	35	Male	32	Bachelors	Full-time	High cholesterol (7)	GP
Vik	43	Male	21	Masters	Full-time	Type 2 diabetes mellitus (>10), costochondritis (3–4)	GP

### Theme 1: A mix of experiences

Participants revealed positive and negative experiences with using Australian PHC services for the management of their chronic illnesses. Mixed experiences with communication, its effect on the quality of care, and negative consequences of inadequate lengths of consultations were described.

#### ‘Everything is at one place’

The participants described the PHC setting as a one-stop shop where ‘everything is at one place’ (Sandy). They valued having other services available in the same clinic, including pathology, radiology, physiotherapy, and dental services. This, according to them, proved to be much more convenient, ‘… and you know the place and the people, so things become easier’ (Sandy), than having to visit different places for different tests for which they had to often wait for at least a week before getting an appointment. They described these experiences as being ‘just easier’ (Shaf) because ‘you don’t need to go to different places’ (Raul).

#### Experiences with treatment and management of chronic illnesses in PHC

The participants presented a mix of experiences with the treatment and management of their chronic illnesses in the PHC setting. All participants were satisfied with their current GPs’ informative communication about the causes of their chronic illnesses, treatment and management regimes, potential side effects of treatments, and reasons for medication changes. Bree was visibly impressed by the ‘very clear’ communication from her GP, especially about ‘what the side effects could be’ of her new medication, as was Pur, who felt his GP was ‘happy to answer questions and was very understanding and very kind about it’, making him feel that his concerns were adequately addressed without any judgement. Dal was impressed with the amount of information and education she was provided with to manage her diabetes with ‘diet, exercise and medication’. When Roi first saw her GP for her knee pain, she really appreciated the level of understanding he showed towards ‘the pain and my concern about what the diagnosis would be’. Sandy also found his GP to be ‘very thorough’ and was glad that he ‘listens and addresses any concerns’.

While participants described positive communication experiences with their current GPs, some had concerns about previous GPs’ poor language skills and limited efforts in building rapport, which affected the quality of care that was provided. Bindu described being ‘sent back with some Panadol’ when she was in a lot of pain and felt some GPs need to show more care and ‘understand when somebody is really in pain’. Bree compared her experiences with doctors in India and Australia and felt that doctors in India were ‘more competent’ than some of those she had seen in Australia. Nee shared similar beliefs about the competency of doctors in India, who she believed were ‘some of the brightest doctors across the globe’ along with the technological advancements within the Indian health care system. She thought that the Australian doctors did not have the same level of expertise and believed that this was due to the ‘vast experience’ Indian doctors gained from the large population in India.

Almost half of the participants felt that some GPs did not provide an adequate amount of time for their face-to-face consultations. They felt ‘rushed’ (Nee) and were often ‘out from the GP’s room within a couple of minutes with a prescription’ (Vik). They stated that they felt like the GP did not want to listen to them. This experience was contrary to the participants’ expectations. They emphasized that the time they spent with their GP during face-to-face consultations provided them with limited opportunities to communicate and discuss their health concerns. Bindu, who was living with multiple chronic illnesses appeared disappointed and described that she expected GPs to listen, understand her issues, and ask questions to try and help treat her, but ‘felt neglected’ instead:

You come in; they don’t even listen to you properly. And then they just give you medication or just send you out. I feel like they don’t have that much time to listen to you. I’m getting the same answer lots of times. I feel like it’s not right, you know, until they don’t examine you. They didn’t check anything. They didn’t ask the questions regarding the situation, and they send you with some medication. I feel like that’s not right. (Bindu)

While participants acknowledged that GPs had many patients to see, they felt it was important that they received adequate consultation time. The participants reported that thorough assessments and examinations were not being conducted during the short consultations and, consequently, their GPs could not understand their symptoms, were dismissive of their concerns and were unable to provide a holistic approach to their care. Sandy described one interaction where he felt his previous GP ‘did not understand’ his concerns about his migraines, ‘started joking’ and wanted to ‘finish the consultation like he has done his part and now you are on your own’. He mentioned that he felt reassured after reading ‘some online resources’. Nee described an experience where she felt her GP was being ‘hasty’ and she was not able to explain her concerns:

I was like, at least give me two minutes, or maybe 3 minutes. Just don’t rush me. And yeah, they just write the prescription, and they’ll be like off you go, scoot now. So yeah, that was not very nice. (Nee)

Study participants confessed that they changed from one GP clinic to another due to these issues:

Sometimes my previous GP was like oh you’re just overthinking or you’re just working really hard. Maybe that’s why this is happening. You have to reduce this or that, you don’t need to worry. But after I think three to four months later you change the GP, go to different one and then you find out no, you’re going through something really serious and you need a treatment for that. (Bindu)

Sandy also preferred ‘not to go’ to his previous GP if his symptoms ‘were not very bad’ and eventually changed to a different GP.

#### Experiences with reception staff

Though not directly involved in the provision of care and management of their chronic illnesses, participants acknowledged the important role of the reception staff in the PHC setting. Bindu described this succinctly, ‘sometimes I feel like receptionists make a big difference. Some of them are really welcoming of you and some of them are just there.’ According to most participants, the reception staff facilitated a homely atmosphere and managed patients with different levels of needs. Bree describes the reception staff at her small GP practice: ‘they knew, you know, every patient by name. They knew good history, so it was quite homely atmosphere’.

However, Nee and Raul described the communication from some reception staff as ‘frustrating’ (Nee) and needing improvement for a compassionate health care experience. According to these participants, the lack of communication about waiting times, especially when this is exceeded beyond acceptable times, added to feelings of stress and disappointment. For example, Nee, who was living with multiple chronic illnesses, expressed her frustration about the reception staff who became ‘annoyed’ when she asked them why there was a delay with her scheduled appointment. Nee explained that the reception staff did not provide a reason for the delay and did not understand that patients also had work commitments and limited time:

The reception is not very vocal in terms of why we have the delay, and they also seem annoyed if you go ahead, and you know if you ask them why is there a delay at all. They do not give you a reason. They’re like, ok, there are seven patients before you, just eight patients before you. But a lot of them like, really seem annoyed. (Nee)

Some reception staff were described as ‘rude’ (Roi), ‘cranky’ (Victor) and lacking empathy and compassion. Nee described her experiences of being treated differently due to perceived ethnic differences:

They’re different in the way they treat, you know, people of the white race and they’re different to the people who are of, let’s say, the Indian race or, you know, some other ethnicity. I think people of the white race get more privilege. This behaviour is more, you know, it’s more predominant in again, receptionists who are white in race. Some of them not all. Some of them are really pleasant. But it does not happen so much with receptionists who are of the Indian cultural background or maybe some other ethnicity. (Nee)

### Theme 2: Facilitators to accessing PHC services in Australia

This theme presents the facilitators in accessing PHC services in Australia as described by the participants. These include the ongoing convenience of telehealth and the benefits of a shared cultural background with that of their GP.

#### Telehealth

While talking about telehealth as an important facilitator of their access to PHC, almost all of the participants made specific reference to the recent global COVID-19 pandemic as the catalyst. During that time, most of the participants explained that they did not need to see their GP in person as much and, therefore, opted for the much-appreciated telehealth services that became increasingly available. Roi stated that after her initial knee examination, she did not need to ‘see him too often’. The option of telehealth consultations proved to be invaluable for the participants during, and post, COVID-19. Nee described telehealth as being ‘really accessible’ and ‘one of the best things to happen’.

Participants described the convenience of having an appointment with their GP over the phone, without having to go in person, for urgent prescriptions, non-urgent blood test results, and consultations that did not require a physical examination. According to them, they did not only save travel and waiting time, but they also received timely care. Nee was happy to not ‘spend time in transporting’ and sitting in the waiting room’ while receiving more prompt’ care through telehealth. For more than half of the participants, telehealth had become the preferred method of consultation when they did not need to see their GP in person. Shaf elaborated on this point.

Unless there’s something I need to show him, I prefer doing it on the phone. I think because he knows my history and, you know, he knows me well, I think it’s more convenient. Unless obviously there’s something I need to physically show him. (Shaf)

Given that almost all of the participants were full-time workers, they appreciated the flexibility of telehealth consultations in seeking health care despite their busy schedules. Telehealth facilitated timely access to PHC services, talk to their GP over the phone, and were able to finish their telehealth consultations within ‘ten to 15 minutes (Nee). According to Roi, with telehealth, there was ‘pretty much no wait time’.

#### Cultural background of GP

The participants established that having a GP from the same cultural background facilitated better communication and a greater level of understanding between them and their GP. Despite living in Australia for over thirty years, Roi and Victor still preferred having a GP of a similar cultural background. These participants felt that the GP was able to provide a greater level of understanding and was perceived to have more of a ‘sympathetic approach’. Similarly, Nee appreciated her GP’s ability to identify potential illnesses that they had experienced within the Indian community:

My current GP is of the Indian background, and he had dealt with so many Indians before so he was able to sort of identify that these are the ailments that I could have. So, from that perspective, I think it was helpful that he was from the Indian background. (Nee)

Having the same cultural background as their GP facilitated better communication by way of similar languages and a greater understanding of dietary requirements. Being able to communicate in their own language was beneficial for participants as they felt that they could explain themselves better and understand the PHC provider more effectively. For instance, Dal appreciated that their GP had some understanding of their food preferences and options, providing a better understanding of the required dietary changes to help control her type 2 diabetes mellitus:

If GP is from the same country or same culture, it’s better he can explain you better. Like I love my food, so it’s very hard to explain to the other GP. I feel so comfortable to explain my current situation more easily. (Dal)

### Theme 3: Barriers to accessing PHC services in Australia

The participants in this study identified unavailability of their preferred GP and waiting times and delays for in-person appointments as barriers that hindered their access to PHC services in Australia.

#### Unavailability of preferred GP

Participants in this study preferred to see a regular GP who supported their health care needs; someone they were able to see when needed and someone who was able to provide them with the care needed to manage their chronic conditions. As Vik described it: ‘a dedicated family GP. However, this proved to be a barrier to accessing timely and relevant PHC services. Bindu found it difficult to get an appointment with her regular GP on several occasions and had to wait for at least one week to be seen: sometimes they’re really busy and you have to wait for a week time, sometimes two weeks’. The inability to access her preferred GP when needed also posed challenges in receiving timely and continued care for Bree. She summarizsed her experience: It was only one doctor on a day so if she was unwell or not coming in, I didn’t have any other option but to go to a completely different person or completely different practice’. Furthermore, some of the participants felt helpless when they reported not getting a GP with specialized expertise in areas such as mental health, Not all the clinics in the locations I’ve been at have had mental health professionals, or then they haven’t had mental health professionals who are free within the next few weeks (Pur).

GPs providing bulk-billed services were preferred by participants and having to pay a co-payment because bulk billing was not available was a significant deterrent. Pur was ‘not bothered’ to contact GPs for an appointment when their website specified that they did not offer bulk-billed services. He also cancelled an appointment after he reached the GP clinic and found out that bulk billing was not an option:

There was one time when I went to a clinic where there wasn’t any specification about bulk billing. Before I went in for my appointment, I found out that there’s no bulk billing. And so, I asked him, if I can, you know, this isn’t what I was expecting. I would like to not go to the appointment, and they cancelled it. (Pur)

He was also disappointed about having to pay the same amount of co-payment for telehealth consultations as others did for face-to-face consultations. He questioned, ‘why am I paying the same amount of co-pay for something that’s done over the phone?. The increasing number of clinics charging a co-payment was a growing concern among participants as they believed that this would limit the number of bulk-billed PHC services, increase PHC costs, and, consequently, reduce access to PHC services:

We know that people will avoid going to the GP because if they have to pay, so that’s something that has to be looked into and it should not be taken lightly by the government. It should be kept as a free service for primary care. (Bree)

#### Long waiting times

Long waiting times to see their GP in-person were identified as a major barrier to seeking PHC. When an appointment was not available, participants had to attend the GPs’ clinics without an appointment and wait between half an hour to three hours. Bindu described having to wait ‘for more than one hour’, even when she was in ‘so much pain’: And you don’t get the appointment, so you have to walk in. But even with an appointment these days, I find it is difficult. You still have to wait longer than your scheduled time’.

Long waiting times deterred participants from returning to the clinic and contributed to their preference for telehealth consultations. Roi stated that she avoided going to the clinic, even when she felt that she needed to see her GP face-to-face, and preferred telehealth ‘“as much as possible now’.

Consistently long waiting times caused a lot of frustration among participants. Waiting for their GP for around an hour every time, even when they had an appointment, affected other aspects of their lives. Raul was frustrated when he summarized this issue:

Everybody here is taking time off from something that they’re doing. Either kids’ pick-up or work or something, and if you’ve got to wait for like an hour every time then you kind of lose one hour just waiting there.

For participants who did not have the option of working from home, ‘almost half a day’ (Nee) was often spent waiting for an appointment to see their GP. Nee stated that she ‘expected a delay of maybe half an hour or 45 minutes’ but became frustrated when she had to wait ‘two hours’ one day and decided to leave ‘the clinic altogether and was like I’ll maybe come another day or go to a different GP’.

## Discussion

Mixed experiences of treatment and management of chronic illnesses within PHC were shared by the participants in this study. This is similar to previous studies that reported on migrants’ experiences with treatment and management of chronic illnesses in PHC settings. Trusting relationships with GPs were found to be significant in chronic disease management among Indian immigrants in the UK (Sidhu *et al*., 2016). Contrasting evidence reported that a lack of concern, rudeness, and poor communication were the experiences of young adult migrants in Greater Western Sydney, Australia (Raymundo *et al*., 2021) and South Asian migrants in Tasmania, Australia (Terry *et al*., 2011). An unapproachable nature of GPs, along with their inattentiveness, contributed to participants losing confidence and feeling discontent with PHC services, leading them to seek PHC service elsewhere (Vakil *et al*., 2023). In the current study, most participants shared positive experiences of receiving compassionate and emotionally supportive care from their GPs, and this facilitated trusting GP-patient relationships. Meanwhile, some participants felt that their concerns were dismissed with poor communication and compassion, resulting in their experiences of sub-optimal care, changes in their GPs and delays in accessing care. Having a mixed health care experience presents the PHC system to the risk of losing the trust of its users.

Having a GP of the same cultural and linguistic background was not a necessity for most participants in this study. It is important, though, to note that in the current study, the participants acknowledged having a GP of the same cultural and linguistic background as a facilitator to better PHC access. They explained that a common culture and language facilitated better communication and understanding between participants and their GP and, in turn, enabled better comprehension of health information. This is similar to previous studies among Asian, African, and Middle Eastern immigrants in Australia that reported the importance of speaking in English to communicate their needs, as well as comprehend their health care providers and educational resources (Khatri and Assefa, 2022; Lakin and Kane, 2023). In both the UK and Canada, language and cultural barriers led to limited knowledge about their chronic illnesses and prevented optimal use of PHC services (Sidhu *et al*., 2016; Rishworth *et al*., 2022), highlighting the preference of GPs with common cultural and linguistic backgrounds.

In this study, issues with untimely access to PHC services and insufficient consultation time were highlighted. Similarly, recent research identified long waiting times as impediments to access for South Asian and other migrants with family and work commitments in Australia, Canada, America and Europe, often leading to limited advice regarding the management and treatment of their chronic illnesses (Rishworth *et al*., 2022; Vakil *et al*., 2023). Statistical evidence from the ABS (2025) also reported longer than acceptable waiting times to get an appointment with a GP for 26% of people aged 15 years and above. Participants in the current study highlighted having to wait unacceptably long times to be seen by their GP for booked appointments, followed by insufficient times given by their GP during the consultations. This is contrary to the situation reported in a study conducted in India, where the maximum amount of time most participants had to sit and wait in the clinic before being seen for their prebooked GP appointment was ten minutes, and they were satisfied with the amount of consultation time provided (Ardey and Ardey, 2015).

The long waiting in the current study is a serious issue given that timely health care is one of the measures suggested by the World Health Organization (2023) for achieving the PHC pillar that focuses on meeting the lifelong health needs of people. An important explanation for the increasing waiting time may be attributed to a shortage of GPs (Australian Medical Association [AMA], 2022). There is growing community demand for GPs, however, GPs are not only opting for fewer working hours, but GP training places are not being filled (AMA, 2022; Behera *et al*., 2022). Extended freezing of Medicare rebates and lack of government support for general practice may be responsible for these shortages, along with a lack of streamlined processes aimed at integrating and registering internationally qualified GPs (AMA, 2022). Freezing of Medicare rebates also means that some GPs are opting for private billing instead of offering bulk-billed consultations. Bulk billing is payment option in Australia, whereby a range of health care services, including GP consultations, are billed to the government, instead of the patient, at the discretion of the service provider (Services Australia, 2024). With private billing, the consultations are billed directly to the patient, sometimes, with the option for the patient to receive a rebate from Medicare (The Royal Australian College of General Practitioners, 2025). Access to Medicare rebates in Australia depends on one’s immigration status, through which the eligibility for Medicare services is determined (Services Australia, 2024) and, therefore, not all immigrants are able to receive rebates after private billing in PHC. The out-of-pocket expenses for these patients reduce accessibility to timely and affordable PHC services.

### Strengths and limitations

From an extensive search of the current literature, it appears that this study may be the first to specifically explore the PHC experiences of Indian immigrants in Australia. However, self-selection of participants in this study presented limitations by way of potential bias and lack of diversity. Most of the participants were middle-aged residents who were highly educated, engaged in full time employment, and spoke fluent English. This presents a lack of diversity and potential bias in the findings as the experiences of younger and older Indian immigrants, students, those with lower levels of education, working in other types of employment or unemployed, and limited English proficiency were not gleaned. Additionally, all participants were from New South Wales, a state with the second largest population of Indian immigrants, and the perspectives of those living in other states were not obtained.

### Recommendations for future research

The participants in this study were highly educated, middle-aged Indian immigrants who spoke fluent English and lived in metropolitan Sydney. However, the Indian diaspora in Australia is a diverse mix of younger, middle-aged, and elderly immigrants with varying levels of education and English fluency and engaged in a range of professions. They also reside in urban and regional areas across Australia. Therefore, further research is needed to gain a better understanding of the PHC experiences of the diverse Indian immigrant population in Australia, including those with financial hardship. Furthermore, it would be beneficial to learn the perspectives of PHC providers for a better understanding of immigrant PHC experiences in Australia, and to provide an insight into the PHC system for immigrants.

### Policy implications

Health policies in Australia aim at improving migrants’ experiences with PHC, however, it is necessary to understand the way they identify themselves as culturally diverse individuals who are shaped by their unique backgrounds and experiences within and beyond their ethnicity. Directing funding to research and PHC services that aim to address these gaps would promote a more holistic approach in providing timely, compassionate, and culturally appropriate care to address the needs and expectations of Indian and other immigrants in Australia.

### Practice implications

Ongoing training on culturally responsive health care for health care providers in PHC setting, including GPs and reception staff, along with more culturally responsive health promotion strategies would enable better access to and quality of PHC.

## Conclusion

This study highlights the importance of timely and compassionate PHC for Indian and other immigrants living with chronic illness in Australia. A commitment to meeting the lifelong health needs of culturally diverse groups requires that the Australian health care system attend to the ongoing untimely access to PHC services. While the findings of this study presented common experiences among Australian immigrants, it is the first study to specifically present those described by Indian immigrants alone.

## Supporting information

10.1017/S1463423626101339.sm001 Singla et al. supplementary material Singla et al. supplementary material

## Supplementary material

The supplementary material for this article can be found at https://doi.org/10.1017/S1463423626101339.

## Table 1. Long description

The table presents detailed characteristics of 11 participants. It includes columns for participant pseudonym, age, gender, years in Australia, education background, employment status, chronic illnesses with time since diagnosis, and primary healthcare service. The participants range in age from 24 to 51 years and have lived in New South Wales, Australia, for periods ranging from 7 to 32 years. Education levels vary from bachelors to masters and PhD candidates. Employment statuses include full-time and part-time. Chronic illnesses listed include endometriosis, chronic back pain, thyroid issues, high blood pressure, type 2 diabetes mellitus, polycystic ovarian syndrome, asthma, mental health issues, and high cholesterol. Primary healthcare services utilized include general practitioners and dietitians.

Navigate back to Table 1..
