# Supplementary material for: Primary health care experiences of Indian immigrants living with chronic illnesses in Australia: a qualitative study
Source: Prim Health Care Res Dev. 2026 Jun 15;27:e63. doi: 10.1017/S1463423626101339 (PMC13280598; doi:10.1017/S1463423626101339)
Supplement: Singla et al. supplementary material [file S1463423626101339sup001.docx]

# **Appendix A**

**Interview Guide**

Date____________________

**Pseudonyms____________________________**

**Background information**

1. What is your current age?
2. Which gender do you identify yourself with?
3. How many years have you been living in Australia?
4. What type of chronic illness or illnesses have you been diagnosed with?
5. How long ago were you diagnosed with it?
6. Where are you receiving health care from for the management and/or treatment? For example, which GP, which specialist, and/or which community health care centre?

**Interview questions**

1. Can you please tell me about your experiences with the care you received from the services we talked about for the illness? This can include your physical or emotional experiences of:
   1. Interactions and communication with health care professionals and other staff
   2. Waiting times
   3. The care you received
   4. The health care environment
   5. Personal observations

Probing questions:

1. What have been some positive experiences (if any)?
2. Have you had any negative experiences?
3. Did the experiences relate to the services or the service provider(s)?
4. Any factors that you feel encouraged you to access these services
5. Any factors that you feel prevented you from accessing these services

**Concluding questions and statements**

1. Is there anything else you would like to add or share about this topic that you feel is important for me to know?
